# Evaluation of *In Vivo* Antidiarrheal Activity of Solvent Fractions of *Hagenia abyssinica* (Rosaceae) in Swiss Albino Mice

**DOI:** 10.1155/2021/8828331

**Published:** 2021-02-13

**Authors:** Zemene Demelash Kifle, Birhanu Berihun Kidanu, Tesfaye Yimer Tadesse, Teshome Fentik Belachew, Seyfe Asrade Atnafie

**Affiliations:** ^1^University of Gondar, College of Medicine and Health Sciences, School of Pharmacy, Department of Pharmacology, Gondar, Ethiopia; ^2^University of Gondar, College of Veterinary Medicine and Animal Sciences, Department of Veterinary Pharmacy, Gondar, Ethiopia; ^3^Debretabor University, College of Health Science, Department of Pharmacy, Debre Tabor, Ethiopia; ^4^Department of Pharmacy, Debre Birhan Health Science College, Debre Birhan, Ethiopia

## Abstract

**Background:**

Ethiopia has several medicinal plants that have been used for their antidiarrheal activity. *Hagenia abyssinica* is the most commonly used medicinal plant for the management of diarrhea in Ethiopia. Thus, this study's aim is to investigate the antidiarrheal effect of solvent fractions of *H. abyssinica*.

**Methods:**

Antidiarrheal activity of extract fractions obtained from different solvents was evaluated by using small intestine transit, enteropooling, and castor oil-induced diarrhea animal models. In all animal models, the solvent fractions treated groups were treated with three different doses (100 mg/kg, 200 mg/kg, and 400 mg/kg) of the solvent fractions, while the negative control group was treated with a vehicle (distilled water), and positive control group was treated with loperamide.

**Results:**

The acute toxicity test revealed that the LD_50_ of *H. abyssinica* is > 2000 mg/kg. In castor oil-induced, the solvent fractions of *H. abyssinica* (at 200 mg/kg and 400 mg/kg) significantly (*P* < 0.05–0.001) prolonged the stool frequency, reduced the weight of feces, and delayed diarrheal onset time as compared with the negative control group. The fractions produced a significant (*P* < 0.05) antimotility effect at the doses of 200 mg/kg and 400 mg/kg as compared to the negative control. All solvent fractions at the middle and higher doses showed a statistically significant dose-dependent reduction in the volume of intestinal contents and weight of the feces. However, the solvent fractions of *H. abyssinica* at a dose of 100 mg/kg failed to produce a statistically significant activity in all parameters (number of wet feces, the onset of diarrhea, and number of total feces) when compared with the negative control group.

**Conclusion:**

The extract fractions obtained from different solvents have shown significant antidiarrheal activity. Thus, this finding supports the claimed traditional use of *H. abyssinica* leaves for the treatment of diarrhea.

## 1. Introduction

Diarrhea is a condition of increased intestinal emptying and increased water content in the stool. Generally, if defecation occurs more than 3 times a day, excretion of fecal with soft/liquid consistency or a combination of both showed an abnormal condition in the defecation process [1]. Eighty percent of the Ethiopian population have depended on traditional medicine due to insufficient access to modern medicine, acceptability by the community, and low cost of herbal medicine [2].

Several medicinal plants that possess antidiarrheal activity are available throughout the world. The antidiarrheal effects of these herbs could be due to the attendance of phytoconstituents including flavonoids, alkaloids, saponins, terpenoids, steroids, and tannins [3]. Scientific investigation for the search on novel antidiarrheal compounds from medicinal plants like leaf extract of *Osyris quadripartite* [4], leaf extract of *Justicia schimperiana* [5], leaf extract of *Myrtus communis* [6], and flower extract of *Ixora Coccinea* [7] have shown promising results.

*H. abyssinica* is the sole species of the genus *Hagenia* and belongs to the family *Rosaceae* [8]. The species is found in different parts of Africa such as Ethiopia, Congo, Tanzania, Uganda, Sudan, Malawi, Burundi, Rwanda, and Kenya [9]. Rosaceae is a large family containing more than 100 genera and 2,000 species of herbs, shrubs, and trees [10]. The bark, flower, leaf, and root parts of *H. abyssinica* have been used for both medical and nonmedical purposes [8]. The ethnobotanical survey revealed that the bark of *H. abyssinica* had been used for the treatment of stomachache, livestock disease, malaria, dermatological conditions, fever, and bronchitis. The flower part of *H. abyssinica* has been used for the management of different diseases such as epilepsy, intestinal worms, evil eye, healing wound, hepatitis, STD's, and biliary disease. The root part of *H. abyssinica* has been used for the treatment of stomachache, cancer, throat disease, and abdominal pain. The leaves of *H. abyssinica* have also been used for the management of cancer, cough, hypertension, livestock disease, bone fracture, wound, and diarrhea [11, 12]. In Ethiopia, the leaf part of *H. abyssinica* has been used for the management of the diarrheal disease [11, 13, 14]. Therefore, this study aims to evaluate the *in vivo* antidiarrheal effect of the leaf's solvent fractions of *H. abyssinica*.

## 2. Materials and Methods

### 2.1. Drugs, Reagents, and Instruments

Activated charcoal (Acura Organics Ltd, New Delhi), loperamide hydrochloride (Brawn Laboratories Ltd, India), castor oil (API, Jordan), Methanol (Blulux, India), ethyl acetate (Blulux laboratories ltd, India), chloroform (Finkem Laboratory Reagent, India), glacial acetic acid, sulfuric acid, ammonia, hydrochloric acid and ferric chloride (BDH Laboratory Supplies Poole, England), benzene (Fisher Scientific, UK), acetic anhydride, ethyl acetate, digital electronic balance (EPH-400 Abron Exports), microhematocrit centrifuge (Medit-Medizin Technik, Germany), Vacuum freeze dryer (Lab freeze group, digital thermometer (Infiniti Medlab Pvt, Ltd., India), Hot air oven (Medit-Medizin Technik, Germany), Germany), qualitative Whatman filters paper No.1, and oral feeding tube were used in this experiment.

### 2.2. Plant Materials

The leaves of *H. abyssinica* were harvested in the Northwest part of Ethiopia, Amhara region of Kosoye, on February 12, 2019. Then the collected leaves of *H. abyssinica* were washed using distilled water and dried at room temperature. The identification and authentication of the leaves of *H. abyssinica* were done by a botanist, and the voucher specimens (003ZDK/2019) were placed at the Department of Biology, University of Gondar, Ethiopia.

### 2.3. Extraction and Fractionation

The leaves of *H. abyssinica* were washed with distilled water to remove dirt and dust, and the cleaned plant materials were dried at room temperature (25–27°C). The plant materials were grounded into a coarse powder with an electrical mill. Therefore, the fine powder plant materials were macerated separately in methanol for roughly 72 hours, and then the plant materials were filtered using Whatman filter paper No. 1. Likewise, a fresh solvent was used to remacerate the marc, filtrates of each successive maceration were concentrated using a rotary evaporator. Lastly, the semidried residues were frozen in a refrigerator and dried using a lyophilizer (Labfreez, China) to entirely confiscate the remaining solvent [15, 16]. Water, ethyl acetate, and chloroform solvents were used for fractionation of the crude extract of *H. abyssinica*. Distilled water was briefly added to the crude extract of *H. abyssinica* and dissolved by using a separating funnel. Chloroform was then added and shaken to dissolve the components. Similarly, on the aqueous layer, an equal volume of ethyl acetate was added to it. In both cases, two layers were separated. The subsequent chloroform and ethyl acetate layers were separated and exposed to evaporation by using a hot air oven (40°C). Then, the dried solvent fractions of *H. abyssinica* were kept separately in a desiccator until being used for the experiment [17].

### 2.4. Preliminary Phytochemical Screening of Leaves Solvent Fractions

Qualitative tests were done for the solvent fractions of *H. abyssinica* for the presence of phytoconstituents such as steroidal compounds, saponins, terpenes, flavonoids, tannins, alkaloids, and phenolic compounds, by procedures as described in Trease and Evans, 1989 [18].

### 2.5. Acute Oral Toxicity Test

The acute oral toxicity of *H. abyssinica* was conducted according to the Organization for Economic Cooperation and Development (OECD) guideline No. 425 [19]. On the first day of the test, one female Swiss albino mice fasted for 4 hrs. Then, 2000 mg/kg of the extract was administered by oral route using oral gavage. The mice were observed for the manifestation of behavioral and physical changes, and special attention was given during the first four hours. Depending on the results from the first mice, the next 4 female mice fasted for an estimated 4 hrs and then a single dose of 2000 mg/kg of the extract was given orally and followed in the same manner. The observation continued daily for a total of fourteen days [19].

### 2.6. Experimental Procedures and Designs

Healthy Swiss albino mice of both sexes weighing 25–30 g were used for this experiment. The mice were retained in a plastic mice cage, with the provision of fed or feed with a standard diet and water. The mice were kept beneath a standard temperature, humidity, and 12 hours light and darkness cycle, and all groups of mice were acclimatized for about 2 weeks before the experiment [20]. Throughout the study period, the mice were assigned into three groups, a positive control group, a negative control group, and a test group containing six mice per group. In all animal models, the first negative control groups received distilled water (10 ml/kg), the second positive control received loperamide (3 mg/kg), and the next three groups (II, III, and IV) received different doses (100 mg/kg, 200 mg/kg and 400 mg/kg) of *H. abyssinica* solvent fractions via the oral route. The doses of the solvent fractions of *H. abyssinica* were determined based on the result of the acute oral toxicity test. The middle doses of the solvent fractions of *H. abyssinica* were 1/10 of the limit dose, the higher doses of the solvent fractions were twice of the middle doses of the solvent fractions of *H. abyssinica,* and a half dose of the middle doses of *H. abyssinica* was the lower dose of the solvent fractions of *H. abyssinica* [19, 21].

### 2.7. Castor Oil-Induced Diarrhea Model

Thirty Swiss albino mice of either sex were deprived of food for 18 hours with free access to water and divided randomly into five groups, as mentioned above. After 1 hour of treatment with the vehicle (distilled water), solvent fractions (100 mg/kg, 200 mg/kg, and 400 mg/kg), and loperamide (3 mg/kg), diarrhea was induced by oral administration of 0.5 ml of castor oil to each mouse. Following their administration, the animals were placed individually into metabolic cages in which the floor was lined with transparent paper for the collection of fecal matter. The transparent paper was changed every hour for a total of 4 hours. The mice were then removed from their cages and the weight of feces was obtained by subtracting the weight of filter paper from the weight of feces and filter paper. The onset of diarrhea, the number of wet stools, the total number, and the total weight of fecal output were noted. Finally, the percentage of diarrheal inhibition, as well as the percentage of the weight of total fecal output, was calculated by using the following formulas [22]:

(1) $$\begin{array}{c} \text{ percentage of diarrheal inhibition}=\frac{\text{mean number of wet stools }\left(\text{control group}-\text{treated group}\right)\text{x }100\;}{\text{mean number of wet stools of the control group}}. \end{array}$$

### 2.8. Castor Oil-Induced Intestinal Transit

Therefore, the experimental mice were fasted for 18 hours and had free access to water. The mice were divided into five groups and treated as described above. After 1 hour of treatment, 0.5 ml of castor oil was administered and 1 ml of 5% activated charcoal suspension in distilled water was administered orally. The mice were then sacrificed by cervical dislocation 2 hours after castor oil administration. Then, the small intestine was dissected out from pylorus to caecum and placed lengthwise on white paper. Finally, the distance traveled by the charcoal meal and the total length of the intestine was measured and expressed as a peristaltic index [23].

(2) $$\begin{array}{c} \text{peristalysisindex }\left(\text{PI}\right)=\frac{\text{distance travelled by the charcoal meal}}{\text{total length of small intestine}}\times 100,\\ \text{\% of inhibition}=\frac{\text{PI of negative control}-\text{PI of drug or extract}}{\text{PI of negative control}}\times 100. \end{array}$$

### 2.9. In Vivo Antidiarrheal Index (ADI)

The ADI for the solvent fractions of *H. abyssinica* was calculated by merging three parameters engaged from the abovementioned models. Then, it was articulated based on the following formula [24]:

(3) $$\begin{array}{c} in\;vivo\text{ antidiarrheal index }\left(\text{ADI}\right)\;=\sqrt[{3}]{\text{Dfreq}\times \text{Gmeq}\times \text{Pfreq}}. \end{array}$$

Dfreq is the delay in defecation time as a percentage of negative control; Pfreq is the reduction in purging frequency in the number of wet stools as a percentage of the negative control; and Gmeq is the gut meal travel reduction as a percentage of negative control).

### 2.10. Castor Oil-Induced Enteropooling Model

Swiss albino mice were deprived of food for 18 hours while water was allowed ad libitum. Then, the mice were grouped and treated similarly as described above. One hour later, 0.5 ml of castor oil was administered to each animal. The mice were sacrificed by cervical dislocation 1 hour after the administration of castor oil; the small intestine of each animal was isolated and weighed. Then, the intestinal content of all mice was collected by draining it into a graduated tube. After the removal of the intestinal content, the intestine of each mouse was reweighed. The volume of each intestinal content was measured [25]. Then, percent reductions in weight and volume of intestinal content were calculated using the following formulas:

(4) $$\begin{array}{c} \text{\% of inhibition by using MVIC}=\frac{\text{MVICC}-\text{MVICT}}{\text{MVICC}}\times 100. \end{array}$$

MVICC is the Mean Volume of Intestinal Content of Control Group; MVICT is the Mean Volume of Intestinal Content of Test Group; and MVIC is the Mean Volume of Intestinal Content).

(5) $$\begin{array}{c} \text{\% of inhibition by using MWIC}=\frac{\text{MWICC}-\text{MWICT}}{\text{MWICC}}\times 100. \end{array}$$

MWICC is the Mean Weight of Intestinal Content of Control Group; MWICT is the Mean Weight of Intestinal Content of Test Group; and MWIC is the Mean Weight of Intestinal Content.

### 2.11. Statistical Analysis

The data obtained from the experiments were expressed as mean ± standard error of means (SEM). Statistical analysis was done using statistical package for social sciences (SPSS) version 24. Between- and within-group analyses were carried out by using one-way ANOVA, and subsequently Tukey's multiple comparison tests. Finally, the findings were considered significant when *P* value < 0.05.

## 3. Results

### 3.1. The Percentage Yield of Plant Material Extraction

At the end of the extraction, 153 (14.6%) grams of dried leaf extract were collected. The yields of the fractions were 47.8%, 29.8%, and 17.5% for the aqueous fraction, ethyl acetate fraction, and chloroform fraction, respectively.

### 3.2. Preliminary Phytochemical Screening of the Solvent Fractions

The phytochemical screening results obtained from the tests were presented in Table 1.

### 3.3. Acute Toxicity Test

In acute toxicity study, leaves extract of *H. abyssinica* revealed no mortality at 2000 mg/kg body weight dose. After administration of the extract, the mice did not show any toxic effects like changes in behavioral activities such as anxiety, polyuria, diarrhea, seizures, and coma. Thus, the leaves extract of *H. abyssinica* at 2000 mg/kg body weight showed good safety and the LD_50_ of the *H. abyssinica* extract is > 2000 mg/kg.

### 3.4. Effects of Solvent Fractions on Castor Oil-Induced Diarrhea

As shown in Table 2, the chloroform solvent fraction and ethyl acetate solvent fraction exhibited a statistically significant reduction in the number of wet feces (*P* < 0.001 for both) and the number of total feces (*P* < 0.05, *P* < 0.01, respectively) at higher doses of the fractions (400 mg/kg) when compared with the negative control group. Similarly, 400 mg/kg doses of the three fractions significantly (*P* < 0.001) delaying the onset of defecation when compared with the negative control group. However, the chloroform fraction and ethyl acetate solvent fractions failed to exhibit a significant effect in all parameters at 100 mg/kg and 200 mg/kg doses as compared to the negative control. Though, the aqueous solvent fraction exhibited a significant effect in all parameters (in the number of wet feces, the onset of diarrhea, and the number of total feces) measured in this model at 200 mg/kg and 400 mg/kg doses when compared with the negative control group. The inhibitions of defecation (%) were 38.46%, 57.91%, and 78.00% by chloroform, ethyl acetate, and aqueous fractions, respectively, at 400 mg/kg of the solvent fractions (Table 2).

### 3.5. Effects on Castor Oil-Induced Intestinal Transit in Mice

The middle and high doses of the solvent fractions of *H. abyssinica* (chloroform, ethyl acetate, and aqueous solvent fractions) significantly suppressed the gastrointestinal motility of charcoal when compared with the negative control group. Similarly, the small intestinal transit was significantly (*P* < 0.001) reduced by Loperamide 3 mg/kg with a percentage value of 67.10%. The reduction of gastrointestinal transit of charcoal (%) was 7.10%, 17.61%, and 31.70% for the chloroform fraction; 14.00%, 35.90%, and 51.92% for the ethyl acetate fraction; and 30.40%, 47.30%, and 58.83% for aqueous fraction at tested doses of 100 mg/kg, 200 mg/kg, and 400 mg/kg, respectively (Table 3).

### 3.6. Effects on Castor Oil-Induced Enteropooling

In the intestinal fluid accumulation test, the weight and volume of the intestinal contents were significantly reduced by the chloroform and aqueous solvent fractions at the tested doses of 200 mg/kg (*P* < 0.01) and 400 mg/kg (*P* < 0.001) when compared with the negative control group. The maximum percentage inhibition of the volume of gastrointestinal contents was detected at 400 mg/kg such as 40.70% (*P* < 0.001), 47.30% (*P* < 0.001), and 52.60% (*P* < 0.001) for aqueous solvent fraction, chloroform solvent fraction, and ethyl acetate solvent fraction, respectively. All fractions exhibited a statistically significant percentage reduction in weight of small intestine with the highest percentage reduction (40.70%, 53.00%, and 47.00%, respectively) at 400 mg/kg dose of the solvent fractions when compared with the negative control group (Table 4).

### 3.7. Antidiarrheal Index

The determination of *in vivo* ADI revealed that the ADI increased with the dose for each fraction. Among the fractions, an aqueous fraction at its higher tested dose had the maximum ADI as compared to 200 mg/kg and 400 mg/kg doses of all fractions, but less than the ADI of Loperamide. The solvent fractions exhibited an ADI of 52.37, 72.17, and 74.14, respectively, for chloroform solvent fraction, ethyl acetate solvent fraction, and aqueous solvent fraction at a dose of 400 mg/kg, respectively, demonstrating a dose-dependent activity on the antidiarrheal index values (Table 5).

## 4. Discussion

Different medicinal plants with antidiarrheal activity have been studied by animal models (effect on gastrointestinal transit, electrolyte, and water secretion) [26, 27]. The antidiarrheal activity of extract fractions obtained from different solvents of *H. abyssinica* has not been investigated. Therefore, the current study was planned to evaluate the antidiarrheal activity of the solvent fractions of *H. abyssinica* via animal models such as castor oil-induced diarrheal model, antipropulsive, and antientropooling.

Castor oil-induced diarrhea is a commonly used method to evaluate the antidiarrheal activity of medicinal plants [28]. The ricinoleic acid that is released from castor oil through the lipase enzyme stimulates irritation in the gastrointestinal mucosa. This irritation caused secretion of platelet-activating factor, nitric oxide, cyclic adenosine monophosphate, prostaglandin, and tachykinins, which are inflammatory mediators. The inflammatory mediators stimulate intestinal motility and increase the secretions of water and some electrolyte. Several studies revealed that castor oil could induce diarrhea within one to two hours following administration of 0.1–0.3 milliliters of castor oil [29, 30].

In the castor oil-induced diarrhea model, the higher doses (400 mg/kg) of all the solvent fractions exhibited a statistically significant activity in all parameters determined: the number of wet and total stools, the weight of wet stools, and the onset of diarrhea. The extract fractions obtained from different solvents could produce their antidiarrheal effect by antisecretory mechanism as it was apparent from the decrease in the total number of wet feces. Moreover, nonsteroidal anti-inflammatory drugs can inhibit castor oil-induced diarrhea apart from its inhibition of prostaglandin synthesis [31]. Likewise, the extract of *H. abyssinica* has also revealed anti-inflammatory activities similar to nonsteroidal anti-inflammatory drugs [32]. Therefore, it is reasonable to suppose that the antidiarrheal activity of *H. abyssinica* solvent fractions might be due to a reduction in the production of prostaglandin. The phytoconstituents like terpenoids have been reported to obstruct the synthesis of prostaglandin [33], which are identified to take part in the activation of gastrointestinal secretions [34]. As a result, the significant antidiarrheal activity observed by the extract fractions obtained from different solvents could be because of the occurrence of different phytoconstituents in the solvent fractions of *H. abyssinica*. The current finding is in line with previous similar studies [4, 35, 36].

The aqueous solvent fraction at its higher dose (400 mg/kg) showed a maximum effect on the percentage inhibition of defecation (78.00%). The chloroform fraction, however, was found to be active either at the middle or at the higher dose. The insignificant activity of the solvent fractions at 100 mg/kg doses could be because of the incapability of the phytoconstituents to reach an adequate amount to elicit antidiarrheal activity. This argument is supported by the fact that activity would be apparent with an increasing dose of the extracts. The current finding is in agreement with previous studies in which the aqueous solvent fractions of numerous medicinal plants have reduced the number of stooling [4, 37].

In the gastrointestinal motility model, the most effective and extensively used antidiarrheal drugs produced their effect by different mechanisms such as by reducing the intestinal motility and blocking the secretion of intestinal contents. The activated charcoal model is employed to investigate the activity of medicinal plants on gastrointestinal motility, and it serves as a marker [5]. The higher doses of the solvent fractions of *H. abyssinica* repressed the transit of charcoal meal or propulsive movement throughout the gastrointestinal tract that demonstrates the solvent fractions of *H. abyssinica* leaves could be able to decrease the frequency of stool. However, the lower doses of all tested doses of the solvent fractions and the middle doses of chloroform fraction and ethyl acetate fraction did not show a statistically significant decrement in the percentage of gastrointestinal motility, and this showed that the solvent fractions have less antimotility effect at the lower and the middle doses of the solvent fractions. Cholinergic activation causes diarrhea by increasing gastrointestinal motility, while anticholinergics prevent diarrhea by inhibiting cholinergic activation [38]. This finding indicates that the solvent fractions have poor anticholinergic activity on gastrointestinal mucosa at the lower and the middle doses of the solvent fractions. The solvent fractions repressed the propulsion of activated charcoal, which showed the efficacy of the extracts in decreasing the vagal peristaltic movements of the gastrointestinal tract system. These also give details about the muscle relaxant activity of the solvent fractions. This pharmacological effect of the solvent fractions might be one of the more likely mechanisms for their antidiarrheal activities.

In the castor oil-induced enteropooling model, all the solvent fractions of *H. abyssinica* significantly decreased the intraluminal fluid accumulation as compared to the negative control group. The current finding agreed with previous similar studies [39, 40]. The maximal activity of the solvent fractions was comparable with the standard drug loperamide, which is one of the most commonly used drugs for the treatment of diarrheal disorder [41]; as presented in the current study, loperamide successfully inhibited the induced diarrhea. The ricinoleic acid, which is the metabolite of castor oil, brings inflammation and irritation of the gastrointestinal mucosa, resulting in prostaglandins secretion. The secreted prostaglandins inhibit the reabsorption of water and NaCl_2_ [42]. Accordingly, the solvent fractions significantly inhibit the gastrointestinal hypersecretion and enteropooling by decreasing gastrointestinal accumulation of fluid or via facilitating reabsorption of water and electrolytes. The antienteropooling effect of the solvent fractions may also be linked to the presence of secondary metabolites such as tannins, steroids, and flavonoids. The phytoconstituents like steroids and flavonoids block the secretion of prostaglandins; by this means, they block the release of prostaglandins and increase the absorption of some electrolytes. Tannins reduce the fluid secretion in the gastrointestinal by different mechanisms such as blocking cystic fibrosis transmembrane conductance regulator and calcium-activated chloride channel, by generating a protein-precipitating reaction to the intestinal mucosa and by free radical scavenging activity [33, 43]. Indeed, it is well established that the liberation of ricinoleic acid from castor oil also causes irritation and inflammation of intestinal mucosa, leading to the release of prostaglandins E2, which results in stimulation of secretion. Similarly, the *in vitro* and *in vivo* experiments have shown that flavonoids, terpenoids, and saponins can decrease the gastrointestinal secretion stimulated by prostaglandins, thereby inhibiting secretion activated by castor oil [44].

Several phytoconstituents obtained from different medicinal plants have shown antidiarrheal activity. Among the phytoconstituents, saponins, triterpenoids, and flavonoids have been reported to prevent gastrointestinal motility and electrolyte secretions [45–47].

*H. abyssinica* contains phytoconstituents such as tannins, phenols, flavonoids, saponins, glycosides, alkaloids, anthraquinones, and terpenoids, as tested by phytochemical screening tests. Therefore, the possible antidiarrheal properties of the solvent fractions of *H. abyssinica* might be due to the abovementioned secondary metabolites.

## 5. Conclusion

The findings of the present study demonstrated that the solvent fractions of *H. abyssinica* possessed significant antidiarrheal activities. The antidiarrheal activities of the solvent fractions could probably be attributed to the presence of phytoconstituents in the *H. abyssinica*. Thus, this finding supports the claimed traditional use of *H. abyssinica* leaves for the management of diarrhea.

## Figures and Tables

**Table 1:** Phytochemical screening of leaves solvent fractions of *H. abyssinica*. (+= Present, - = absent).

Metabolites	Ethyl acetate fraction	Chloroform fraction	Aqueous fraction
Tannins	+	_	+
Alkaloids	_	_	+
Saponins	+	_	+
Flavonoids	+	+	+
Triterpenoid	+	_	_
Phenols	+	+	+
Steroids	_	+	_
Glycosides	+	_	+

**Table 2:** Antidiarrheal effects of solvent fractions of *H. abyssinica*.

Group	Onset of diarrhea (min)	Number of wet feces	Number of total feces	% inhibition of defecation
Control	46.83 ± 3.21	9.10 ± 0.47	11.83 ± 0.60	—
CF 100 mg/kg	60.83 ± 2.12b^*∗*^	8.30 ± 0.34b^*∗∗∗*^	10.00 ± 0.72b^*∗∗∗*^	8.80
CF 200 mg/kg	72.83 ± 3.38b^*∗∗∗*^	7.60 ± 0.46b^*∗∗∗*^	8.60 ± 0.46b^*∗∗∗*^	16.50
CF 400 mg/kg	102 ± 2.47 a^*∗∗∗*^b^*∗*^	5.60 ± 0.23a^*∗∗∗*^b^*∗*^	7.20 ± 0.20a^*∗*^b^*∗∗∗*^	38.46
Loperamide 3 mg/kg	154.66 ± 6.8a^*∗∗∗*^	1.40 ± 0.2a^*∗∗∗*^	3.00 ± 0.25a^*∗∗∗*^	84.60

Control	44 ± 2.84	9.10 ± 0.47	10.00 ± 0.85	—
EAF 100 mg/kg	55.5 ± 2.64b^*∗∗∗*^	8.16 ± 0.77b^*∗∗∗*^	9.00 ± 0.85b^*∗∗∗*^	10.32
EAF 200 mg/kg	68.00 ± 3.65b^*∗∗∗*^	7.10 ± 0.83b^*∗∗∗*^	7.00 ± 0.77b^*∗∗∗*^	22.20
EAF 400 mg/kg	99.00 ± 4.15a^*∗∗∗*^b^*∗∗∗*^	3.83 ± 0.34a^*∗∗∗*^b^*∗∗∗*^	5.33 ± 0.49a^*∗∗*^b^*∗*^	57.91
Loperamide 3 mg/kg	141.00 ± 7.14a^*∗∗∗*^	1.66 ± 0.21a^*∗∗∗*^	3.10 ± 0.32a^*∗∗∗*^	76.62

Control	48.50 ± 4.52	9.10 ± 0.47	10.16 ± 0.75	—
AF 100 mg/kg	71.33 ± 8.76b^*∗∗∗*^	5.16 ± 0.47b^*∗∗∗*^	6.33 ± 0.71b^*∗∗∗*^	43.29
AF 200 mg/kg	88.65 ± 7.74a^*∗*^b^*∗∗∗*^	3.50 ± 0.42a^*∗∗*^b^*∗*^	5.50 ± 0.61a^*∗∗*^	61.53
AF 400 mg/kg	91.57 ± 10.88a^*∗∗*^b^*∗*^	2.00 ± 0.25a^*∗∗∗*^	3.66 ± 0.33a^*∗∗∗*^	78.00
Loperamide 3 mg/kg	138.50 ± 7.65a^*∗∗∗*^	1.83 ± 0.33a^*∗∗∗*^	3.60 ± 0.18a^*∗∗∗*^	80.00

Data are expressed as mean ± SEM (*n* = 6); analysis was performed with One-Way ANOVA followed by Tukey test; ^a^compared to negative control; ^b^compared to loperamide 3 mg/kg; ^*∗*^*P* < 0.05, ^*∗∗*^*P* < 0.01, and ^*∗∗∗*^*P* < 0.001; CF: chloroform fraction; EAF: ethyl acetate fraction; AF: aqueous fraction; negative controls received 10 ml/kg distilled water.

**Table 3:** Effects of solvent fractions on castor oil-induced gastrointestinal motility in mice.

Dose administered	Length of small intestine (cm)	Distance moved by the charcoal meal (cm)	Peristaltic index (%)	% inhibition
Control	58.30 ± 1.02	46.50 ± 1.14	84.82 ± 1.47	—
CF 100 mg/kg	58.16 ± 1.24	46.83 ± 1.44^b^^*∗∗∗*^	80.53 ± 1.67^b^^*∗∗∗*^	7.10
CF 200 mg/kg	57.16 ± 1.93	40.83 ± 2.41^b^^*∗∗∗*^	71.43 ± 3.14^a^^*∗*^^b^^*∗∗∗*^	17.61
CF 400 mg/kg	56.83 ± 0.94	33.83 ± 1.86^a^^*∗∗*^^b^^*∗∗∗*^	59.50 ± 3.92^a^^*∗*^^b^^*∗∗∗*^	31.70
EA 100 mg/kg	55.33 ± 1.49	41.00 ± 3.04^b^^*∗∗∗*^	74.50 ± 4.72^a^^*∗*^^b^^*∗∗∗*^	14.00
EA 200 mg/kg	55.83 ± 1.04	31 ± 2.61^b^^*∗∗∗*^	55.54 ± 4.24^a^^*∗*^^b^^*∗∗∗*^	35.90
EA 400 mg/kg	56.38 ± 1.62	23.50 ± 1.47^a^^*∗∗∗*^^b^^*∗∗*^	41.68 ± 2.57^a^^*∗∗∗*^^b^^*∗∗*^	51.92
AF 100 mg/kg	56.60 ± 1.23	34.16 ± 1.66^b^^*∗∗∗*^	60.35 ± 3.56^b^^*∗∗∗*^	30.41
AF 200 mg/kg	56.16 ± 0.96	25.66 ± 2.57^a^^*∗∗∗*^^b^^*∗∗*^	45.69 ± 4.22^a^^*∗∗*^^b^^*∗∗*^	47.34
AF 400 mg/kg	57.00 ± 1.45	20.33 ± 1.23^a^^*∗∗∗*^^b^^*∗*^	35.66 ± 3.37^a^^*∗∗∗*^^b^^*∗*^	58.83
Loperamide 3 mg/kg	55.83 ± 0.87	18.66 ± 0.88^a^^*∗∗∗*^	33.00 ± 1.52^a^^*∗∗∗*^	59.80

Data are expressed as mean ± SEM (*n* = 6); analysis was performed with One-Way ANOVA followed by Tukey test; ^a^compared to negative control; ^b^compared to loperamide 3 mg/kg; ^*∗*^*P* < 0.05, ^*∗∗*^*P* < 0.01, and ^*∗∗∗*^*P* < 0.001; CF: chloroform fraction; EAF: ethyl acetate fraction; AF: aqueous fraction; negative controls received 10 ml/kg distilled water.

**Table 4:** Effects of solvent fractions on castor oil-induced enteropooling in mice.

Dose administered	Mean volume of small intestinal content (gm)	% inhibition	Mean weight of small intestinal content (ml)	% inhibition
Control	0.76 ± 0.04	—	0.81 ± 0.01	—
CF 100 mg/kg	0.69 ± 0.02^b^^*∗∗∗*^	9.20	0.69 ± 0.018^a^^*∗*^^b^^*∗∗∗*^	14.80
CF 200 mg/kg	0.58 ± 0.02^a^^*∗∗*^^b^^*∗∗∗*^	23.60	0.6 ± 0.02^a^^*∗∗*^^b^^*∗∗∗*^	26.00
CF 400 mg/kg	0.40 ± 0.04^a^^*∗∗∗*^	47.30	0.48 ± 0.02^a^^*∗∗∗*^	40.70
EA 100 mg/kg	0.62 ± 0.21^a^^*∗*^^b^^*∗∗∗*^	18.40	0.6 ± 0.02^a^^*∗∗*^^b^^*∗∗∗*^	26.00
EA 200 mg/kg	0.53 ± 0.09^a^^*∗∗*^^b^^*∗*^	30.20	0.51 ± 0.02^a^^*∗∗∗*^^b^^*∗*^	37.00
EA 400 mg/kg	0.36 ± 0.14^a^^*∗∗∗*^	52.60	0.38 ± 0.01^a^^*∗∗∗*^	53.00
AF 100 mg/kg	0.68 ± 0.018^b^^*∗∗∗*^	10.50	0.71 ± 0.01^a^^*∗*^^b^^*∗∗∗*^	12.30
AF 200 mg/kg	0.55 ± 0.02^a^^*∗∗*^^b^^*∗∗∗*^	27.60	0.59 ± 0.01^a^^*∗∗*^^b^^*∗∗∗*^	27.10
AF 400 mg/kg	0.45 ± 0.08^a^^*∗∗∗*^	40.70	0.43 ± 0.02^a^^*∗∗∗*^	47.00
Loperamide 3 mg/kg	0.36 ± 0.02^a^^*∗∗∗*^	52.60	0.4 ± 0.01^a^^*∗∗∗*^	50.60

Data are expressed as mean ± SEM (*n* = 6); analysis was performed with One-Way ANOVA followed by Tukey test; ^a^compared to negative control; ^b^compared to loperamide 3 mg/kg; ^*∗*^*P* < 0.05, ^*∗∗*^*P* < 0.01, and ^*∗∗∗*^*P* < 0.001; CF, chloroform fraction; EAF: ethyl acetate fraction; AF: aqueous fraction; negative controls received 10 ml/kg distilled water.

**Table 5:** *In vivo* antidiarrheal index of solvent fractions of *H.abyssinica* leaves.

Dose	Delay in defecation (time of onset in min, Dfreq) (%)	Gut meal travel distance (Gmeq) (%)	Purging frequency in a number of wet stools (%)	*In vivo* ADI
DW 10 ml/kg	—	—	—	—
CF 100 mg/kg	29.89	7.10	8.80	12.31
CF 200 mg/kg	55.51	17.61	16.5	25.26
CF 400 mg/kg	117.80	31.7	38.46	52.37
EAF 100 mg/kg	26.13	14.00	10.32	15.57
EAF 200 mg/kg	54.54	35.90	22.2	35.16
EAF 400 mg/kg	125.00	51.92	57.91	72.17
AF 100 mg/kg	47.07	30.40	43.29	39.54
AF 200 mg/kg	82.78	47.30	61.53	62.22
AF 400 mg/kg	88.80	58.83	78.00	74.14
Loperamide 3 mg/kg	185.56	59.80	82.57	97.13

DW = distilled water, CF = chloroform fraction, EAF = ethyl acetate fraction, AF = aqueous fraction, and ADI = antidiarrheal index.

## Data Availability

The data sets used and/or analyzed during the current study are available from the corresponding author upon reasonable request.
